# Alcohol-Seeking Behavior: The Roles of the Hypothalamic-Pituitary-Adrenal Axis and the Endogenous Opioid System

**Published:** 1998

**Authors:** Christina Gianoulakis

**Affiliations:** Christina Gianoulakis, Ph.D., is a research scientist at the Douglas Hospital Research Centre and an associate professor in the Department of Psychiatry and the Department of Physiology, McGill University, Montreal, Quebec, Canada

**Keywords:** AOD use behavior, hypothalamus-pituitary axis, pituitary-adrenal axis, endogenous opioids, physiological stress, AOD craving, self medication, cortisol, adrenocorticotropic hormone, corticotropin RH, endorphins, dopamine, reinforcement, hereditary factors, AOD use susceptibility, animal model, literature review

## Abstract

Both the hormones of the hypothalamic-pituitary-adrenal (HPA) axis and the endogenous opioid system are activated in response to stress as well as after alcohol consumption, supporting the hypothesis that stress can influence both alcohol consumption and craving for alcohol. Activation of the HPA axis by stress or alcohol results in the production of glucocorticoid hormones, such as cortisol. Those hormones, in turn, are important for the release of the brain chemical dopamine in certain brain areas that are associated with the rewarding and reinforcing effects of alcohol and other drugs. Alcohol-induced release of certain endogenous opioids similarly results in dopamine release in those brain regions. Through this mechanism, both the HPA axis and the endogenous opioid system may influence alcohol consumption. Consequently, genetically determined differences in the activities of the HPA axis and endogenous opioid system may help determine a person’s alcohol consumption level and vulnerability to alcoholism.

Both genetic and environmental factors play significant roles in determining alcohol consumption. Stress is one environmental factor that may influence the initiation and continuation of heavy drinking. For example, in studies with humans, increased levels of anxiety and stress were associated both with high alcohol consumption and with relapse to heavy drinking by abstinent alcoholics (Hore 1971; Kushner et al. 1990). Similarly, de Wit (1996) found that stressful life events, as well as onetime (i.e., acute) reexposure to alcohol, caused abstinent alcoholics to experience increased desire for alcohol and to relapse to drinking.

Studies in laboratory animals confirmed the associations between stress, reexposure to alcohol, and restored alcohol-seeking behavior. The studies were performed with rats that previously had learned to press a lever to obtain alcohol but had been abstinent for a period of time (Lê et al. 1998). When the animals were subjected to a stressful situation (e.g., a mild electrical shock to their feet) or received a single alcohol injection, they resumed their previous alcohol-seeking behavior (i.e., pressing a lever to obtain alcohol). The researchers concluded that both stress and a priming alcohol injection could reinstate alcohol-seeking behavior in animals. This situation is similar to inducing alcohol craving and relapse to heavy drinking in abstinent human alcoholics.

The mechanisms underlying the relationship between stress and alcohol consumption are not well understood. Some researchers have suggested that alcohol consumption relieves anxiety and, as a result, helps people cope with stressful situations (Pohorecky 1991). The evidence for the anxiety- and stress-reduction hypothesis, however, is mixed, because experiments have demonstrated that alcohol can both reduce and produce anxiety (Pohorecky 1981). Nevertheless, several studies have strongly supported the notion that stress influences alcohol consumption by humans (Pohorecky 1991).

Although more studies are needed to better understand how stress modulates alcohol consumption, biological systems that are affected by both alcohol and stress most likely play an important role. One such system is the hypothalamic-pituitary-adrenal (HPA) axis; another is a group of brain chemicals (i.e., neurotransmitters) called the endogenous opioid system. This article briefly describes the HPA axis and endogenous opioid system and their responses to alcohol and stress. It then reviews the roles of those responses in mediating the rewarding and reinforcing effects^1^ of alcohol. Finally, the article discusses how genetic differences in the HPA axis and endogenous opioid system may increase a person’s risk for developing alcoholism.

## The HPA Axis

**Figure 1:**
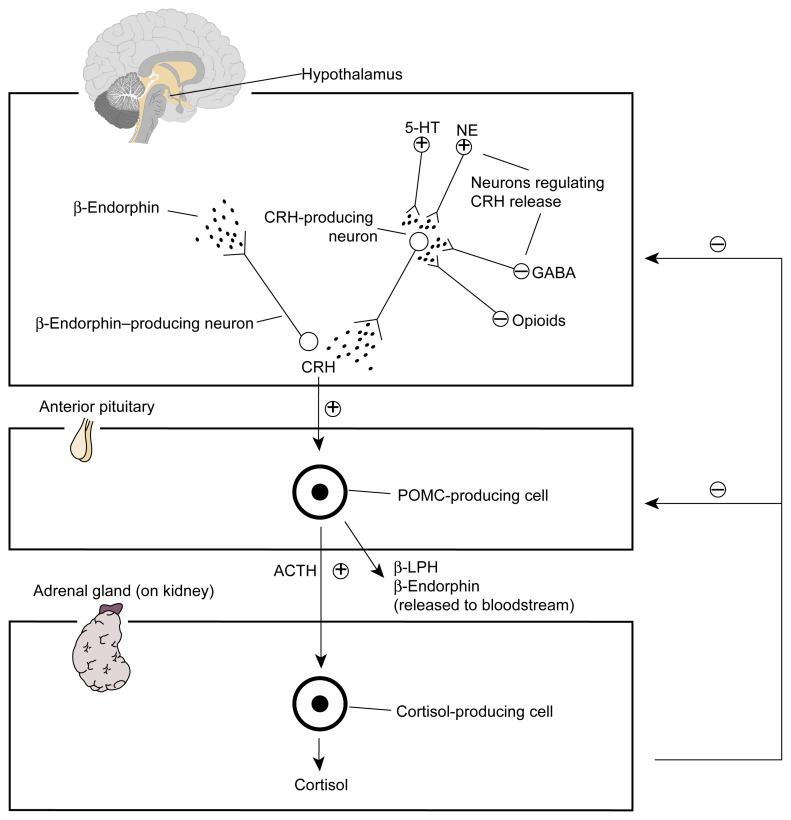
The major components of the stress response. Both alcohol and stress can induce nerve cells in one brain region (i.e., the hypothalamus) to produce and release corticotropin-releasing hormone (CRH). Within the hypothalamus, CRH stimulates the release of a hormone that produces morphinelike effects (i.e., β-endorphin). CRH also is transported to a key endocrine gland, the anterior pituitary gland. There, CRH stimulates production of a protein called proopiomelanocortin (POMC). POMC serves as the basis for a number of stress-related hormones, including adrenocorticotropic hormone (ACTH), β-lipotropin (β-LPH), and β-endorphin. ACTH stimulates cells of the adrenal glands to produce and release the stress hormone cortisol. When cortisol levels reach a certain level, CRH and ACTH release diminishes. Other neurons releasing serotonin (5-HT), norepinephrine (NE), gamma-aminobutyric acid (GABA), or endogenous opioids also regulate CRH release. NOTE: ⊕ excites; ⊝ inhibits.

The HPA axis is a hormone system that plays a central role in the body’s stress response. As the name implies, this axis involves hormones that are produced (i.e., synthesized) in the brain’s hypothalamus and anterior pituitary gland^2^ as well as in the adrenal glands—small, hormone-producing structures located atop the kidneys. This system, which controls a wide variety of metabolic functions, is activated in response to all kinds of stress, both physical and psychological. The major stress hormones of the HPA axis include the following (see figure 1, p. 204):

- Corticotropin-releasing hormone (CRH), which is produced in the hypothalamus
- Adrenocorticotropic hormone (ACTH), which is synthesized in the anterior pituitary gland
- Cortisol,^3^ a glucocorticoid hormone that is produced in the adrenal glands.

Activation of the HPA axis induces certain nerve cells (i.e., neurons) in the hypothalamus to release CRH. Specific blood vessels (i.e., the hypothalamic-hypophyseal portal vessels) carry the hormone to the pituitary gland, specifically the anterior lobe. There, CRH interacts with molecules (i.e., receptors) located on the surface of special hormone-producing cells. (For more information on hormone-receptor interactions, see the article by Hiller-Sturmhöfel and Bartke, pp. 153–164.) This interaction stimulates ACTH synthesis in those cells and its release into the bloodstream. ACTH then is transported to cells in the adrenal glands that have receptors for the hormone on their surface. As a result, those cells begin to produce and release cortisol and other glucocorticoids. Cortisol is transported through the blood to numerous organs throughout the body, where it induces physiological stress responses (e.g., increases in blood sugar levels and breakdown of proteins and fat molecules).

Increased cortisol concentrations in the blood trigger a decrease in CRH and ACTH release through a mechanism known as negative feedback. In addition, several neurotransmitter systems modulate CRH production and release in the hypothalamus. Some neurotransmitters (e.g., serotonin and norepinephrine) stimulate CRH release, whereas other neurotransmitters (e.g., gamma-aminobutyric acid [GABA] and endogenous opioids) inhibit CRH release.

## The Endogenous Opioid System

Endogenous opioids are small protein molecules (i.e., peptides) chemically related to morphine and heroin that are produced primarily in the pituitary gland and brain.^4^ They are involved in various physiological processes, such as pain relief (i.e., analgesia); euphoria; and the rewarding and reinforcing effects of various drugs, including alcohol (Akil and Cicero 1986). Three distinct families of opioid peptides exist: endorphins, enkephalins, and dynorphins. A single large protein (i.e., precursor protein) gives rise to each family type.^5^ Thus, the protein proopiomelanocortin (POMC) is the precursor of all endorphins, proenkephalin gives rise to the enkephalins, and prodynorphin is processed into the dynorphins (Akil and Cicero 1986). The most potent endogenous opioid is β-endorphin.

**Figure 2:**
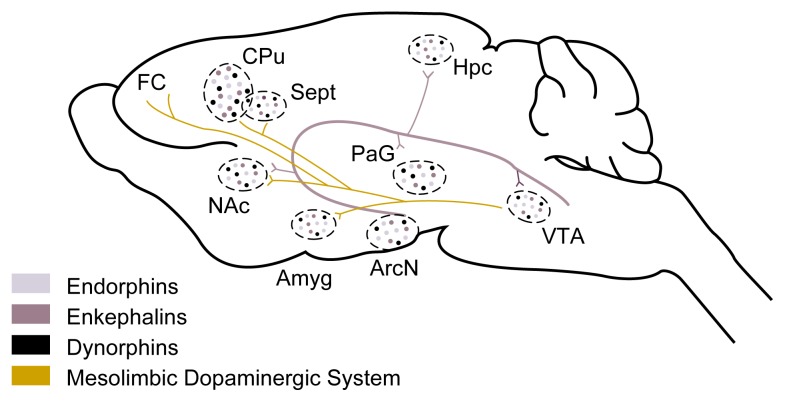
Lengthwise view of the rat brain showing the brain regions in which certain stress hormones (i.e., endogenous opioids) are released. Those hormones—endorphins (light purple), enkephalins (purple), and dynorphins (black)—and the brain chemical (i.e., neurotransmitter) dopamine are involved in the processes of reward and reinforcement. Endorphin-producing nerve cells are located primarily in the arcuate nucleus (ArcN); they extend to and release endorphin in various brain areas (purple). Nerve cells in several regions produce enkephalins and dynorphins, which may be released either in the same region or in distant regions through networks of nerve cells (not shown). A nerve-cell network called the mesolimbic dopaminergic system (gold line) carries dopamine from the ventral tegmental area (VTA) to various parts of the brain. NOTE: Amyg = amygdala; CPu = caudate putamen; FC = frontal cortex; Hpc = hippocampus; NAc = nucleus accumbens; PaG = periaqueductal grey area; Sept = septum.

Not all endogenous opioids are produced in the same brain regions (see figure 2, p. 206). The enkephalin-producing neurons are distributed widely throughout the brain, with the highest concentrations found in the nucleus accumbens (NAc) and the hypothalamus. Similarly, dynorphin-producing neurons are found in various brain regions, including the caudate nucleus, NAc, and septum. (For a summary of the functions of these and other brain regions mentioned in this article, see table, p. 205.)

Endorphin-producing neurons, in contrast, are located mainly in one section of the hypothalamus called the arcuate nucleus. Nevertheless, endorphins can affect numerous brain regions, because the parts of the neurons that release endorphins and other chemical messengers (i.e., the axons) can extend to other brain areas, including the amygdala, NAc, septum, ventral tegmental area (VTA), and various parts of the hypothalamus. Some of those brain regions play important roles in mediating the rewarding and reinforcing effects of alcohol and other drugs (AODs). The endorphins then modulate the activity of adjacent neurons in the brain regions in which they are released.

Like most hormones and neurotransmitters, endogenous opioids alter the activities of their target cells by interacting with specific receptors on the surface of those cells. Three major classes of opioid receptors exist: mu (μ), delta (δ), and kappa (κ). The three classes of endogenous opioids differ in the strength (i.e., affinity) with which they bind to the various receptors. Thus, β-endorphin binds to both μ and δ opioid receptors with equivalent affinity. Enkephalins bind most strongly to δ opioid receptors, whereas dynorphins have high affinity for κ opioid receptors (Charness 1989).

### The Relationship Between ACTH and β-Endorphin

**Figure 3:**
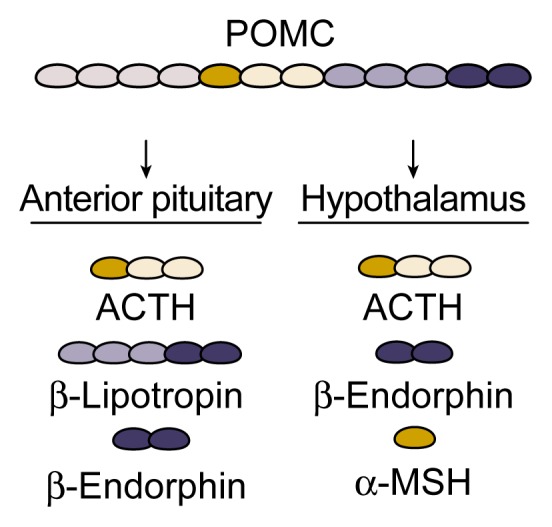
Schematic representation of hormones derived from proopiomelanocortin (POMC) in the hypothalamus and anterior pituitary gland, brain areas fundamental to hormone production and regulation. POMC, which itself is inactive, is cut into smaller, active hormones in a process called posttranslational processing. The products of this process differ in the hypothalamus and pituitary gland. The major products in the hypothalamus are β-endorphin, α-melanocyte–stimulating hormone (α-MSH), and small amounts of adrenocorticotropic hormone (ACTH). The major products in the anterior pituitary are ACTH, β-lipotropin, and β-endorphin.

Although the HPA axis and the β-endorphin system have distinct functions within the body, the two systems are structurally related in that the β-endorphin precursor POMC also serves as a precursor for ACTH. POMC, which is produced primarily in the pituitary gland and in the arcuate nucleus of the hypothalamus, is processed to yield several hormones, including ACTH and β-endorphin (see figure 3, p. 207). To add further complexity to this process, the hormones derived from POMC differ depending on where they are produced. Thus, POMC processing in the hypothalamus yields β-endorphin, α-melanocyte–stimulating hormone (α-MSH), and small amounts of ACTH. In the anterior pituitary gland, however, POMC generates ACTH, β-lipotropin, and β-endorphin. All those products are stored together in bubblelike structures (i.e., secretory granules) within the cell and are usually released together in response to various stimuli, including stress and AODs (Guillemin et al. 1977).

## Responses of the HPA Axis and the β-Endorphin System to Stress and Alcohol

Both the HPA axis and the β-endorphin system are activated in a similar fashion when the organism is exposed to physical or psychological stress or to alcohol. Activation of the HPA axis occurs immediately following the onset of stress, when nerve fibers from several brain regions interact with the CRH-producing neurons in the hypothalamus to induce increased CRH release. Similarly, alcohol ingestion results in increased CRH release from the hypothalamus. CRH then is transported to the anterior pituitary, where it stimulates ACTH release into the bloodstream. ACTH, in turn, induces the release of cortisol from the adrenal gland.

Both stress and alcohol ingestion also stimulate the β-endorphin system. Thus, ACTH release from the anterior pituitary also leads to the release of β-endorphin, which is stored in the same secretory granules as ACTH in anterior pituitary gland cells. Furthermore, CRH induces elevated β-endorphin release from the axons of endorphin-producing neurons in the hypothalamus. As mentioned previously, those axons extend to other brain regions, such as the NAc and VTA, which are associated with the rewarding and reinforcing effects of AODs, and the periaqueductal grey area, which is involved in analgesia. The increased β-endorphin release may induce a calming and relaxing effect as well as analgesia, thereby helping the person to cope with or adapt to the stressful situation.

**Table:** Functions of Some Brain Regions

Brain Region	Proposed Functions*
Amygdala	Emotion and memory
Caudate nucleus	Control of voluntary movements
Frontal cortex	Processes of planning and decision making
Hippocampus	Learning and memory
Hypothalamus	Coordination of hormonal and behavioral aspects of affect and emotion, including eating and drinking behavior
Limbic system	Learning, memory, and emotion; acts as the link between the higher cognitive functions and more primitive emotional responses; Includes the amygdala, hippocampus, septum, and hypothalamus and their interconnecting fibers
Nucleus accumbens	Reinforcement of behavior and attention
Periaqueductal grey area	Pain relief (i.e., analgesia); contains opiate-sensitive cells
Ventral tegmental area	Arousal and reinforcement of behavior

* Many functions are controlled by the interaction of numerous brain regions.

## Roles of the HPA Axis and β-Endorphin System in Controlling Alcohol Consumption

As described in the previous section, acute alcohol consumption activates the HPA axis and β-endorphin release. In addition, the stress hormones of the HPA axis, such as CRH and cortisol, as well as endogenous opioids—particularly β-endorphin—have been implicated in the control of alcohol consumption.

### The HPA Axis

The role of the HPA axis in modulating AOD consumption has been investigated in laboratory animals. For example, Fahlke and colleagues (1995) studied rats whose adrenal glands had been removed and who consequently could not produce the glucocorticoid corticosterone. Following removal of the adrenal glands, the animals exhibited reduced alcohol consumption. When the animals were treated with corticosterone, however, they resumed their alcohol consumption. These findings suggest that glucocorticoids help control alcohol ingestion.

Other studies have suggested that glucocorticoids influence AOD consumption by acting on a group of neurons in the VTA, called the mesolimbic dopaminergic system (see figure 2). The axons of those neurons, which release the neurotransmitter dopamine, extend to the NAc, frontal cerebral cortex, amygdala, and septum—brain regions associated with mediating the rewarding and reinforcing effects of AODs (Koob et al. 1998). Researchers found that glucocorticoids may increase the activity of those dopamine-releasing neurons through various mechanisms (Piazza and Le Moal 1996). Interestingly, animals who easily become addicted to AODs experience a greater increase in dopamine release after being exposed to a stressful situation; moreover, the extent of dopamine release depends on the extent of the animals’ corticosterone secretion (Piazza and Le Moal 1996). These observations suggest that stress-induced glucocorticoid secretion represents a hormonal mechanism through which stressful experiences stimulate the activity of the mesolimbic dopaminergic system, thereby enhancing an individual’s vulnerability to AODs (Piazza and Le Moal 1996). For example, one could speculate that once the stressful event is terminated and the stress-induced activity of the HPA axis and dopamine secretion decline, the person experiences a very mild withdrawal reaction. Consequently, the individual might desire to drink to maintain the activity level of those hormonal and neuronal systems.

Another hormone activated by stress is CRH, which activates the HPA axis by stimulating ACTH release from the pituitary and, subsequently, glucocorticoid release from the adrenal glands. Substances that block the effects of CRH (i.e., CRH antagonists), thereby preventing activation of the HPA axis, have been shown to prevent stress-induced relapse to drug-seeking behavior in rats (Erb et al. 1998). In addition, CRH is found in brain regions other than the hypothalamus, such as the central nucleus of the amygdala. Increased CRH release in that nucleus has an anxiety-inducing effect (Koob and Britton 1996). During alcohol withdrawal, CRH release outside the hypothalamus is enhanced and may cause the anxiety that often occurs in people undergoing withdrawal. This hypothesis is supported by findings that administration of a CRH antagonist outside the hypothalamus can reverse withdrawal-induced anxiety (Koob and Britton 1996). Thus, CRH may play an important role in AOD consumption, although the hormone’s exact role is not well understood.

### The β-Endorphin System

In contrast to CRH and glucocorticoids, the role of the endogenous opioids, particularly β-endorphin, in alcohol-seeking behavior is better understood. β-Endorphin’s actions also involve the mesolimbic dopaminergic system. Experimental evidence has suggested that dopamine release in the NAc from the cells of the mesolimbic dopaminergic system may contribute, at least in part, to the rewarding effects of many drugs of abuse (Koob et al 1998). Benjamin and colleagues (1992) demonstrated that alcohol administration in rats increases dopamine release in the NAc. Moreover, by giving the rats naltrexone (an agent that interferes with the action of endogenous opioids) before administering alcohol, the researchers prevented the alcohol-induced dopamine release. Those findings suggest that the endogenous opioid system may help mediate alcohol’s rewarding effects by modifying the activity of the mesolimbic dopaminergic system.^6^

**Figure 4:**
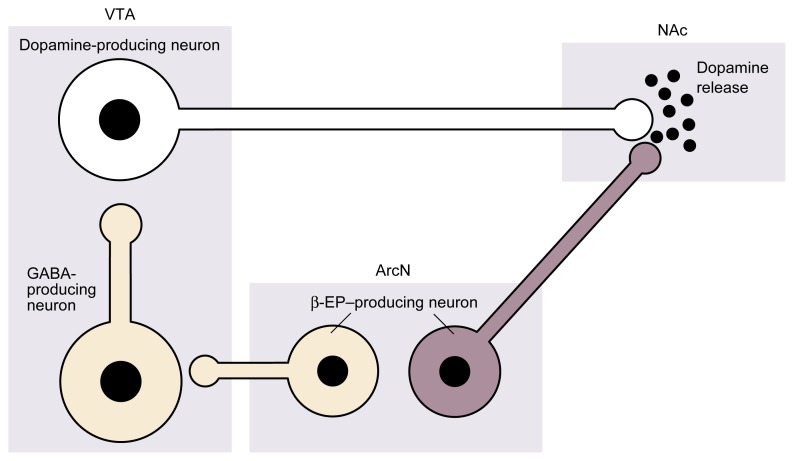
Schematic representation of the possible influence of β-endorphin (β-EP) on dopamine release in the nucleus accumbens (NAc). Dopamine is a brain chemical (i.e., neurotransmitter) involved in reward and reinforcement processes, and the NAc is a brain region involved in mediating alcohol’s positive reinforcing effects. β-EP is produced in the arcuate nucleus of the hypothalamus (ArcN) by nerve cells (i.e., neurons) that extend to other brain regions, including the ventral tegmental area (VTA) and the NAc. β-EP can stimulate dopamine release in the NAc through two mechanisms. First, it can interfere with (i.e., inhibit) neurons in the VTA that produce gamma-aminobutyric acid (GABA), a neurotransmitter that normally inhibits the dopamine-producing neurons in the VTA. Inhibition of GABA production leads to increased dopamine production and release in the NAc. Second, β-EP can directly stimulate (i.e., excite) dopamine-producing neurons in the NAc. Alcohol stimulates β-EP release in both the VTA and NAc. Purple structures indicate excitatory mechanisms, and gold structures indicate inhibitory mechanisms.

Jamensky and Gianoulakis (1997) have developed a model to explain the relationship between alcohol administration, dopamine release in the NAc (Benjamin et al. 1992), and β-endorphin release in the VTA and NAc (Rasmussen et al. 1998). According to the model, alcohol stimulates β-endorphin–producing neurons in the hypothalamus to increase β-endorphin release in the NAc and/or VTA. The β-endorphin, in turn, can directly or indirectly stimulate dopamine release in the NAc (see figure 4, p. 208). For example, β-endorphin released in the VTA acts on neurons that produce the neurotransmitter GABA. GABA normally inhibits the activity of the dopamine-producing neurons in the VTA. β-Endorphin released in the VTA, however, blocks the activity of the GABA-producing neurons. As a result, those neurons can no longer inhibit the dopamine-producing neurons in the VTA, and dopamine is released into the NAc. In addition, β-endorphin may stimulate dopamine release in the NAc by interacting directly with μ and δ opioid receptors on the axons of the dopamine-producing neurons.^7^

### Dynorphins

Dynorphins also can interact with certain opioid receptors (i.e., κ receptors) on the axons of dopa xmine-producing cells. In contrast to β-endorphin, however, this interaction blocks dopamine release and may therefore induce aversive rather than pleasurable effects (Spanagel et al. 1992). Accordingly, it appears likely that agents which increase the interaction of dynorphins with κ receptors on dopamine-producing cells may decrease the reinforcing effects of AODs and thereby prevent the development of AOD dependence. This hypothesis is supported by the observation that compared with alcohol-preferring mice, those mice that avoid alcohol have higher levels of κ opioid receptors, of the genetic molecules (i.e., mRNA) that produce the dynorphin precursor prodynorphin, and of various dynorphins in the NAc (Jamensky and Gianoulakis 1997).

## Genetic Differences in the Activities of the HPA Axis and Endogenous Opioids

If components of the HPA axis and the endogenous opioid system can modulate alcohol-seeking behaviors or the rewarding and reinforcing effects of alcohol, then genetic factors that influence the activities of those components might help determine a person’s alcohol consumption levels. The following sections explore the evidence for this hypothesis.

### The Role of Genetic Differences in the HPA Axis

Increased HPA activity in response to various stimuli generally is reflected by increased cortisol levels in the blood. Animal studies consistently have demonstrated that the activity of the HPA axis increases following alcohol exposure (Pohorecky 1981). Most studies in humans, however, have not detected significant changes in blood cortisol levels after alcohol ingestion (Gianoulakis et al. 1996). The absence of a cortisol response to alcohol may result from the fact that the alcohol concentrations used in those studies generally were low (i.e., 0.25 to 0.75 g/kg [gram alcohol per kilogram body weight], corresponding to approximately one to three standard drinks^8^). Increased cortisol levels have been observed, however, in humans who had consumed greater amounts of alcohol (Schuckit et al. 1987).

Because the presence of cortisol in the blood appears to be needed for alcohol consumption in certain animal models (Fahlke et al. 1995), genetically determined differences in the response of the HPA axis to stress or alcohol might predispose an individual to heavier alcohol consumption. Indeed, animal studies have shown that animals exhibiting greater increases in blood cortisol levels in response to a stress challenge also consume greater amounts of alcohol (Higley et al. 1991). As with humans, animals respond differently to a stress challenge. Some animals become very active and show great increases in corticosterone and dopamine levels (i.e., are high responders), whereas other animals are less active and show smaller increases in corticosterone and dopamine levels (i.e., are low responders). High responders also demonstrate greater self-administration of drugs (e.g., amphetamines, cocaine, and opioids) than do low responders, supporting the hypothesis that greater stress-induced increases in corticosterone and dopamine play a role in enhanced vulnerability to the addictive properties of AODs (Higley et al. 1991). Consistent with this hypothesis, removal of the adrenal glands, which prevents an increase in corticosterone levels, also decreased the alcohol intake of alcohol-preferring rats to the levels of non-alcohol-preferring rats (Piazza and Le Moal 1996). The addition of corticosterone to the animals’ drinking water reversed the effects of adrenal gland removal.

### The Role of Genetic Differences in the β-Endorphin System

Similar to genetic differences in the HPA axis, genetic differences in the activity of components of the endogenous opioid system (i.e., opioid peptides and opioid receptors) may influence alcohol consumption. For example, elevated release of β-endorphin or enkephalins in response to alcohol in the VTA and/or NAc might induce increased dopamine release in the NAc, thereby enhancing alcohol’s reinforcing effects and promoting alcohol consumption. Similarly, genetic factors could lead to a higher density of μ and δ opioid receptors in the VTA and/or NAc, creating more opportunities for interactions between endogenous opioids and those receptors. As a result, greater activation of the dopamine-producing neurons would occur, leading to increased dopamine release. Increased dopamine release, in turn, might promote alcohol’s reinforcing effects and alcohol consumption.

Experimental evidence indicates that differences in the endogenous opioid system, such as those described in the previous paragraph, exist between animals with different alcohol-preference levels. For example, a study comparing mice of an alcohol-preferring strain and mice of an alcohol-avoiding strain found differences consistent with a genetically determined hormonal role in the preference for alcohol (Jamensky and Gianoulakis 1997). Specifically, the study determined the following results:

- The alcohol-preferring mice exhibited greater β-endorphin release from the hypothalamus, both before and after alcohol administration.
- The alcohol-preferring mice had a higher density of δ opioid receptors (to which β-endorphin and enkephalins bind, leading to enhanced dopamine release) and a lower density of κ opioid receptors (to which dynorphins bind, resulting in reduced dopamine release) in the NAc.
- The alcohol-preferring mice exhibited lower levels of prodynorphin mRNA and dynorphins in the NAc.

Each of those conditions could lead to increased stimulation of the dopamine-producing neurons and more pronounced dopamine release in response to alcohol in the NAc of the alcohol-preferring mice. Those effects might contribute to alcohol’s reinforcing effects and be at least partially responsible for the greater alcohol preference exhibited by those animals.

Genetically determined differences in the β-endorphin response to alcohol also have been found in young adult humans at high and low risk for the future development of alcoholism (Gianoulakis et al. 1996). Thus, after consuming one to three standard drinks, high-risk individuals (i.e., those with a family history of alcoholism dating back at least two generations) exhibited greater β-endorphin release from the pituitary gland than did low-risk individuals (i.e., those without a family history of alcoholism). Three mechanisms might help explain a potential association between enhanced pituitary β-endorphin release in response to alcohol and higher alcohol consumption in high-risk people:

- Increased pituitary β-endorphin release may be associated with a pronounced increase in β-endorphin release in various brain regions.
- β-Endorphin released from the pituitary into the bloodstream may enter the brain in low, but probably sufficient, quantities to help initiate alcohol’s reinforcing effect.
- Alcohol itself may alter the blood-brain barrier^9^ so that β-endorphin released from the pituitary into the blood may enter the brain more easily and exert its pleasurable effects.

## Summary

The studies reviewed in this article have demonstrated that one-time alcohol ingestion in both humans and experimental animals may stimulate the release of endogenous opioids in both the brain and the rest of the body. Thus, the body may respond to alcohol as if the person had ingested a small quantity of an opioid drug (Gianoulakis and de Waele 1994). The enhanced opioid activity may initiate alcohol’s positive reinforcing effects either indirectly, by activating the mesolimbic dopaminergic system, or directly, through an as yet unknown pathway. The pleasurable effects of alcohol, in turn, may elicit or enhance alcohol-seeking behavior and craving for alcohol, maintain drinking, and eventually even lead to alcoholism.

Similar to alcohol, stress also stimulates the release of endogenous opioids, which may induce a calming effect and help the individual to cope with the stressful situation. Indeed, stress and alcohol initiate similar hormonal responses, such as activation of the HPA axis and the endogenous opioid system. Consequently, alcohol administration before a stressful situation can initiate the hormonal response that allows an individual to cope with and overcome the stressful situation. In other words, ingestion of small amounts of alcohol can biochemically prepare a person to cope with subsequent stress.

The use of alcohol as a form of “self-medication” to manage stress, however, is complicated by the fact that alcohol also exerts numerous pharmacological effects besides the stress response. For example, high alcohol doses usually have sedative, sleep-inducing (i.e., hypnotic), depressing, or anxiety-inducing effects. Furthermore, frequent alcohol use can lead to the development of tolerance to and physical dependence on alcohol. In alcohol-dependent people, alcohol’s initial, anxiety-reducing effect is short lived and followed by a period of increased anxiety, the extent and duration of which depends on the amount of alcohol consumed and the duration of alcohol dependence. Thus, alcohol consumption to relieve anxiety and stress is often unsuccessful, becomes less effective with prolonged drinking, and is associated with a risk of developing alcohol dependence with its associated social and medical problems. Therefore, alcohol should not be used as a self-medication to relieve depression or stress-associated anxiety.

Genetic differences in the body’s hormonal responses to stress and alcohol ingestion may exist between people. Those differences likely play an important role in determining a person’s sensitivity to alcohol’s pleasurable effects, level of craving for alcohol, and extent of vulnerability to excessive drinking and alcoholism. Future studies aimed at elucidating the biological mechanisms responsible for individual differences in the vulnerability to alcohol’s addictive properties are needed. For example, such studies might investigate whether enhanced activation of the HPA axis and increased cortisol release following stress or alcohol consumption or an increased sensitivity to cortisol’s effects (e.g., in activating dopamine release) influence a person’s vulnerability to alcoholism. Additional studies should determine whether the biological mechanisms underlying the vulnerability to excessive alcohol consumption differ among different people or different animal models of alcoholism. Such investigations may allow researchers to determine the specific biological factors contributing to the vulnerability to alcoholism in each individual and may lead to the development of new and more effective treatments for alcoholism.

## Footnotes

1 The term “rewarding effects” refers to alcohol’s actions on the brain that make the drinker “feel good” (i.e., induce pleasurable effects). The term “reinforcing effects” refers to alcohol’s actions on the brain that make the drinker seek even more alcohol.

2 The anterior pituitary gland, which is located below the brain and which is attached to the hypothalamus, is not considered part of the brain, because it is a hormone-producing (i.e., endocrine) gland and does not consist of nerve cells.

3 Cortisol is the primary glucocorticoid in humans. In rodents, which are frequently used in animal studies of the HPA axis, the primary glucocorticoid is corticosterone.

4 Endogenous opioids are also produced in smaller amounts in other organs (i.e., the periphery), including the heart, placenta, pancreas, kidneys, and gastrointestinal organs.

5 Like many other proteins, endogenous opioids are not produced directly in their final form. Instead, cells often generate large, nonfunctional precursor molecules that are then cut by specific enzymes into one or more smaller and functional proteins. This process is known as “posttranslational processing.”

6 If the endogenous opioid system mediates, at least in part, alcohol’s reinforcing effects, then naltrexone would block those reinforcing effects. Accordingly, people who take naltrexone while consuming alcohol would not experience alcohol’s reinforcing effects, and eventually the alcohol amount they consume as well as their craving for alcohol would decline. This process, which probably underlies naltrexone’s effect in reducing craving and alcohol consumption in recovering alcoholics, is called extinction.

7 Enkephalins may similarly stimulate dopamine release in the NAc.

8 One standard drink is defined as one 12-ounce can of beer or wine cooler, one 5-ounce glass of wine, or 1.5 ounces of distilled spirits.

9 The blood-brain barrier is an anatomic-physiological feature of the brain that prevents or slows the passage of many chemicals or disease-causing organisms from the blood into the brain.
